# Nitrogen Adsorption Measurement for Pore Structure Characterisation of Cement–Oil Shale Ash Composite Exposed to an Aggressive Salt Environment

**DOI:** 10.3390/ma19040772

**Published:** 2026-02-16

**Authors:** Regina Kalpokaitė-Dičkuvienė

**Affiliations:** Laboratory of Materials Research and Testing, Lithuanian Energy Institute, Breslaujos 3, LT-44403 Kaunas, Lithuania; regina.kalpokaite-dickuviene@lei.lt

**Keywords:** sulfate attack, metakaolin, ash, pore size distribution, cement, calcium silicate hydrate, C–S–H, NAD, chloride attack

## Abstract

Despite cement remaining a dominant material in the construction industry, researchers are increasingly exploring strategies to reduce its consumption by incorporating supplementary cementitious materials or by developing alternative binder systems utilising various ashes produced by power plants during the combustion of different waste streams. In this context, the present study investigates the influence of two types of oil shale ash on the pore structure of C–S–H under aggressive environmental conditions. To address these issues, a comprehensive pore structure analysis was conducted using nitrogen gas physisorption, applying multiple analytical approaches including Dubinin–Radushkevich, Horvath–Kawazoe, quench solid density function theory, and Barett–Joyner–Halenda for pore volume and pore size distribution. Pore surface fractal dimension obtained by Neimark Kiselev and Frenkel–Halsey–Hill was compared. The results revealed that the deterioration of C–S–H structure depends on the ash type and the exposure duration to the sulfate–chloride solution.

## 1. Introduction

Cement remains a dominant material in the construction industry; however, its production generates substantial carbon dioxide emissions [1]. Consequently, researchers are increasingly exploring strategies to reduce cement consumption by incorporating supplementary cementitious materials or by developing alternative binder systems through the utilization of various ashes produced in power plants during the combustion of different waste streams [2,3,4].

The properties and durability of alternative cementitious materials depend strongly on their microstructure and pore size, ranging from macro- to nanoscale [5]. The hydration of Portland cement systems results in the formation of various hydration products, with calcium silicate hydrate (C–S–H) being the most important among others [6]. Due to its non-crystalline structure and intermixture with other hydration phases, the analysis of C–S–H, and primarily its porosity, has been a topic of study for decades [7,8,9]. The porosity of C–S–H comprises interlayer space (around 1 nm), gel pores (1–3 nm), interhydrate pores (around 10–12 nm), and small capillary pores (larger than 12 nm) [8]. According to IUPAC, these pores can be classified as micropores (with pore width less than 2 nm) and mesopores (pore width between 2 and 50 nm) [10].

Exposure to sulfate and chloride ions originating from soil, groundwater, and marine environments can adversely affect cement-based materials by degrading their pore network, leading to strength deterioration [11,12,13,14]. The exposure to aggressive environmental conditions, such as cryogenic and salt solutions, induces changes in C–S–H pore structure, which can be evaluated by employing different methods [15,16,17,18]. For example, the deterioration of C–S–H pore structure caused by freeze–thawing was evaluated by applying two methods: mercury intrusion porosimetry (MIP) and low-field nuclear magnetic resonance (LF-NMR) [15]. An impact of sulfate or chloride ions on the C–S–H structure was indicated by scanning electron microscopy equipped with electron dispersive spectroscopy (SEM-EDS) [19,20,21]. In samples exposed to low-pH sulfate solution, an increase in pore volume larger than 10 nm, leading to the loose C–S–H structure formation, was measured by nitrogen physisorption (NAD) [18].

Generally, experimental techniques and methods for pore structure analysis can be categorized into two groups: indirect and direct methods [5,22]. The direct methods are mostly non-invasive or non-destructive, producing images for pore size and shape evaluation [5]. In indirect methods, the detection of the response of the tested probe provides information about pore parameters [5,23,24,25,26]. Some experimental techniques are expensive, while others require well-trained personnel; therefore, MIP and gas- or water vapour physisorption techniques remain the most popular. Their theoretical methods used for the interpretation are well established and enable the assessment of pore size from less than 12 nm in size [22,23,25]. The main difference between nitrogen physisorption (NAD) and water vapour sorption (WVS) applications lies in the assessment of the interlayer space, which is possible mainly by water vapour [22,24]. However, more recently, R. Kurihara and I. Maruyama concluded [24] that the NAD technique can quantitatively evaluate more than 70% of C–S–H gel pores, while the surface area of gel pores measured by NAD and H NMR are highly comparable, thus confirming the high reliability of the NAD method.

The accuracy of the obtained results and the interpretation of microstructural changes in the C–S–H structure by the NAD method depends on the sample preparation parameters [9,22,27]. For example, R. Kurihara and I. Maruyama pointed out that the fineness of the sample affects the surface area measured by NAD [9]. Z. Zhang and G.W. Scherer compared the impact of nitrogen drying, oven drying, freeze drying, supercritical drying, and an isopropanol replacement method on preserving the texture of C–S–H using the NAD measurement technique [27]. It was demonstrated that oven drying was the worst method for the pore structure in the range of 2–10 nm [27]. On the contrary, the isopropanol replacement method preserves the C–S–H microstructure without an influence on portlandite [27].

Pore size distribution, pore volume, and surface area determination from the physisorption isotherms are based on applying a proper theoretical model [22,23,25]. The classical method based on the macroscopic Kelvin equation, such as BJH (Barret–Joyner–Halenda), is the most common method used to characterize cementitious materials [5,7,18,20,27,28]. On the contrary, microscopic methods based on density functional theory (DFT) are more reliable than BJH, providing a comprehensive pore analysis in the microporosity range [7,22] and accurately representing the C–S–H structure. The application of both DFT and BJH methods has been found in a few studies [29,30,31,32]; meanwhile, other analytical methods, such as the Dubinin–Radushkevich (DR) and Horvath–Kawazoe (HK) approaches, are rare.

The application of shale ash for partial cement replacement was discussed in a series of scientific papers [4,33,34,35,36,37,38]. Research has primarily examined the microstructural characteristics and mechanical properties of cementitious composites incorporating oil shale ash; however, their long-term durability, with a focus on the C–S–H pore structure, remains insufficiently explored. The impact of two types of oil shale ash on the properties of blended cementitious materials subjected to freeze–thawing was analysed in the author’s previous papers [37,38]. Results revealed that the origin and microporosity of the ash significantly alter the volume of pores (less than 6 nm) in the cement-based composite [37]. It was demonstrated that binder mineralogy strongly influences the durability of cementitious materials when cement is partially replaced with metakaolin and carbonate-rich oil shale ash due to the formation of a refined pore structure with increased volumes of reaction products, primarily C–S–H [38]. However, the impact of long-term curing in aggressive environments on C–S–H porosity remains undiscussed.

Since the NAD technique was indicated as a reliable method for C–S–H pore structure analysis [24], it was used for a comprehensive analysis of blended cementitious compositions under the impact of an aggressive solution in this study. More than 25% of the cement was partially replaced by two types of oil shale ash, intermixed with pozzolanic material (metakaolin), and subjected to long-term exposure in an aggressive solution. The aim was to obtain information on changes in the microporosity range by applying rarely used DR and HK methods. Mesoporosity was analysed by comparing data obtained from the DFT and BJH methods, while the Neimark–Kiselev (NK) and Frenkel–Halsey–Hill (FHH) methods were employed to assess pore surface roughness, thereby deepening the understanding of the complexity of the C–S–H gel structure affected by aggressive environmental conditions.

## 2. Materials and Methods

Throughout the study, two batches of blended cement pastes were prepared, with cement replaced by 20% oil shale ash and 6% metakaolin, at a water-to-binder (w/b) ratio of 0.5 by weight. Such a high cement replacement level enabled the evaluation of the impact of different oil shale ashes on the microstructural changes of the cementitious paste. Metakaolin, a pozzolanic material, was incorporated to activate hydration reactions in the stabilisation of ettringite and the production of monocarboaluminate. The detailed phase analysis in blended (oil shale ash/metakaolin/cement) systems is provided in the author’s previous study [38]. Ash No. 1 and No. 2 were taken from Esti and Auvere power plants, located in Estonia, respectively [37,38]. The main difference among ashes is that the microporosity of ash No. 2 is higher than that of ash No. 1 [37]. The chemical composition of cement, two types of oil shale ashes and metakaolin are presented in detail in the previous author’s studies [37,38]. Two batches of paste samples, denoted MA1 and MA2, were prepared for the test and hydrated in lime water for 28 days to prevent additional carbonation and ensure consistent results among samples. Notations of samples include: metakaolin, ash No. 1 or No. 2, curing duration and medium (Table 1).

After 28 days, one set of samples, referred to as the control, was left in lime water (tap water saturated with Ca(OH)_2_), while the other set was immersed in an artificial seawater solution, consisting of 3% (NaCl) and 5% (Na_2_SO_4_) solution. Such an artificial solution was used by other researchers as well [13,39,40,41]. The volumetric ratio of the solution to the sample was equal to 25. The pH of the fresh solution was 7.6 ± 0.1 and was monitored daily with a pH meter. A total of 5% H_2_SO_4_ sulfuric acid was used to adjust the pH value of the solution to approximately 8. The solution was renewed every week during the first month and monthly until the end of the test.

**Figure 1:**
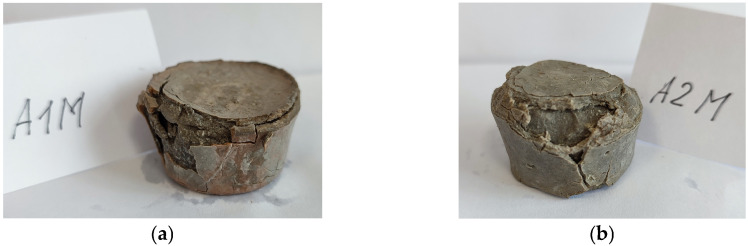
MA1 sample on the left (**a**) and MA2 sample on the right (**b**) after long-term exposure in salt solution.

Samples (5 cm in diameter and 3 cm in height) were collected for analysis and visual inspection from both liquid environments after 3 and 7 months. Visual deterioration of the external surface was seen after 3 months of exposure, while after 7 months, samples were severely damaged (Figure 1). Since decalcification of C–S–H takes place after long-term exposure [12,39], the central part of the samples was taken for the analysis.

Details on the sample drying with isopropanol and preparation for gas physisorption measurements can be found in [32]. A gas sorption test was conducted on a Autosorb iQ-K/MP (Quantachrome, Boynton Beach, FL, USA) analyser with a built-in degassing station operating up to 350 °C. The relative pressure (*p*/*p*_0_) ranges from 10^−7^ to 0.999, and pore size ranges from 0.35 to 200 nm. NAD measurements were performed on dried, degassed (2 h at 60 °C) samples using nitrogen as the adsorbate. The specific surface area, micro–mesopore size distribution and volume were obtained by applying different methods on isotherms, including Brunauer, Emmett and Teller (BET), Dubinin–Radushkevich (DR), Horvath–Kawazoe (HK), Barett–Joyner–Halenda (BJH) and quenched solid density functional theory (QSDFT) approach. Pore surface roughness was analysed by fractal dimension calculation using the Frenkel–Hasley–Hill (FHH) and Neimark–Kiselev (NK) methods.

## 3. Results and Discussion

### 3.1. Sorption Isotherms

**Figure 2:**
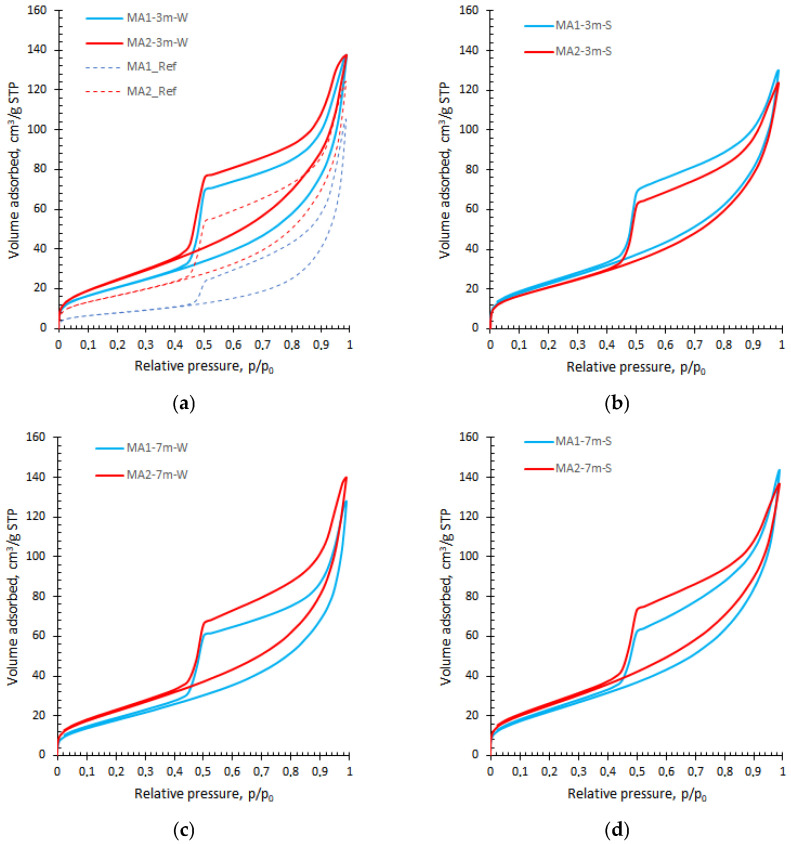
Adsorption/desorption isotherms of MA1 and MA2 samples cured in lime water (**a**,**c**) and salt (**b**,**d**) for three and seven months.

The nitrogen adsorption/desorption isotherms of all samples are presented in Figure 2, and they are very similar to those obtained by others researchers [9,22,31]. According to the recommendations of the International Union of Pure and Applied Chemistry (IUPAC), there are six types of isotherms with or without a hysteresis loop, and uptake at very low *p*/*p*_0_ [23,42]. Figure 1 shows that all the isotherms are close to type IV, typical of mesoporous materials, although they do not exhibit the final saturation plateau. The absence of a saturation plateau indicates the presence of a network of macropores (less than 100 nm) or mesopores, which are not completely filled with pore condensate [42].

A gradual curvature at very low (*p*/*p*_0_ < 0.1) indicates a transition from mono- to multilayer adsorption; however, due to the inhomogeneity of surfaces, the adsorption of multilayer may start earlier than the monolayer is formed, resulting in the overlapping of the monolayer [42].

The hysteresis loop between adsorption and desorption branches in the multilayer adsorption range provides information about the shape of the pores [42]. The loops with the step-down desorption branch have distinctive features of type H5, suggesting the presence of partially blocked and open mesopores [42]. On the other hand, they are close to type H2(b), indicating a wide distribution of independent pores that differ in pore (with the same neck) or neck size (with the same pore width) [42].

The comparison of the isotherms shows that longer exposure to lime water increased the volume of adsorbed gas (Figure 2a), which did not change significantly after subsequent exposure to water or salt solution (Figure 2c,d). Nevertheless, the hysteresis loops narrow with increasing exposure time, especially for the MA1 sample, thus suggesting the pore interconnectivity effect.

### 3.2. Mesopore Volume and Size Analysis

The BJH method, widely used for pore size analysis, is based on the macroscopic Kelvin equation, where pores are assumed to be cylindrical in shape [23]. However, broad hysteresis indicates the presence of ink-bottle pores of different pore or neck size. Therefore, the microscopic QSDFT approach is more appropriate, as it is based on statistical mechanics and accounts for pore wall heterogeneity, thereby enabling the analysis of micropores in addition to mesopores [23].

**Figure 3:**
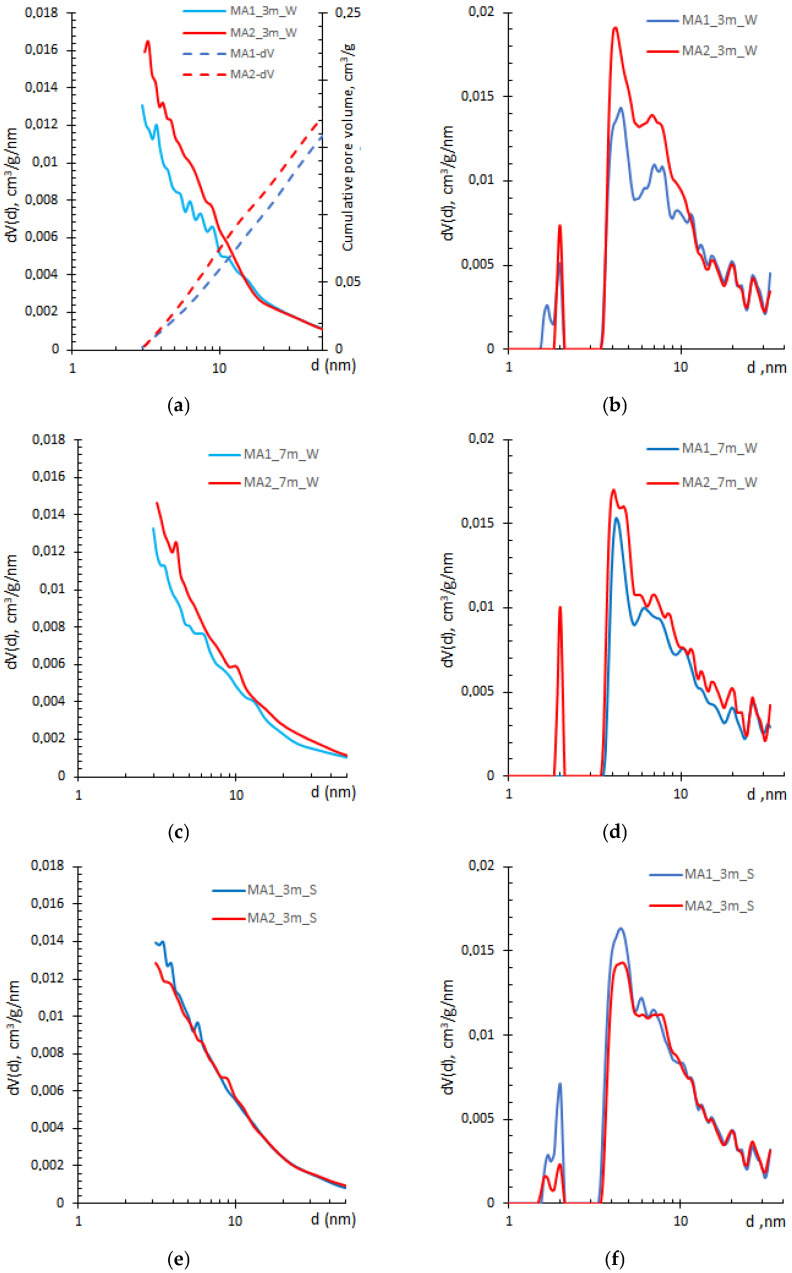

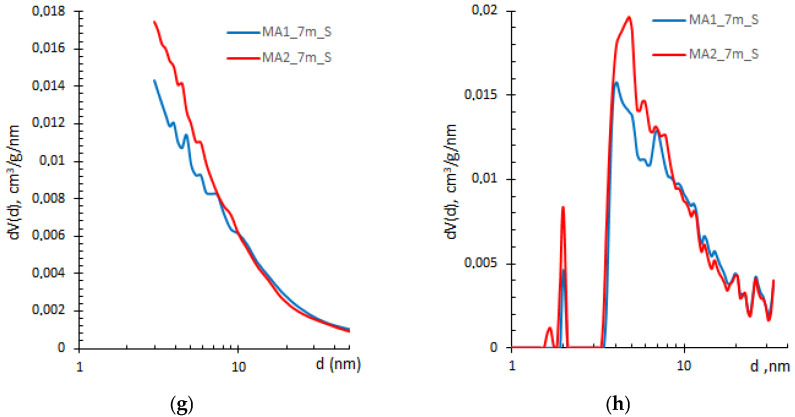
BJH (**a**,**c**,**e**,**g**) and QSDFT (**b**,**d**,**f**,**h**) pore size distribution curves for MA1 and MA2 samples cured in lime water and salt for three and seven months.

It is worth mentioning that the cumulative pore volumes obtained by the BJH method (illustrated by dashed curves “MA1(2)-dV” in Figure 3a) are similar across all samples, indicating the method is applicable in the mesoporosity range. It agrees well with the findings presented in [22,27,30].

The hybrid (slit/cylinder) pore shape model, giving the lowest fitting error, was used to evaluate the pore size distribution by QSDFT method. Curves obtained by both methods, shown in Figure 3, yield quite different results. The QSDFT approach provides more detailed information than the BJH method on pore size up to 10 nm, especially for the MA2 samples. While BJH shows no distinctive peaks, a few peaks at around 2 nm, 4 nm, and 6 nm are observed in the QSDFT curves for both compositions. Due to pozzolanic reactions, the filling of pore space by additional hydration products [38] reduces these peaks after prolonged exposure to lime water. The reduced total pore volume, accompanied by an increase in average pore size, suggests reduced pore accessibility or the formation of closed porosity in the MA1 sample (Table 2). However, the MA2 sample shows an increase in pore volume at 2 nm, even though the peaks at 4 nm and 6 nm are reduced. This behaviour could be attributed to greater pore interconnectivity, as the total pore volume remains nearly unchanged while the average pore diameter increased more than 10% (Table 2).

After exposure to a salt solution for 3 months, the specific surface area of the MA1 samples increased, whereas the MA2 composition decreased (Table 2), suggesting different reaction rates or damaging microstructural effects between the compositions. The pore size distribution remains trimodal for both compositions, though the volume of pores larger than 5 nm increased (Figure 3f). Longer exposure in an aggressive environment tends to increase the total pore volume and formation of a more refined pore structure, especially in the MA2 samples (Table 2).

The pore connectivity determines the penetration of aggressive ions and the leaching of dissolved ions. In contrast, the formation and deposition of reaction products (ettringite and gypsum) lead to the deterioration of the pore structure [11,12]. According to the previous study [38], both compositions, MA1 and MA2, contain a large amount of portlandite (CH), unconsumed by MK, thus increasing the availability of Ca ions needed for the expansive phase formation. Therefore, the further total pore volume increment with the enhancement of pores in the range up to 10 nm indicates the refined pore structure, which is more likely associated with the deterioration of C–S–H structure, driven by C–S–H decalcification [12,39], rather than the sulphate ingress blocking effect mentioned in [11].

### 3.3. Micropore Volume and Size Analysis

**Figure 4:**
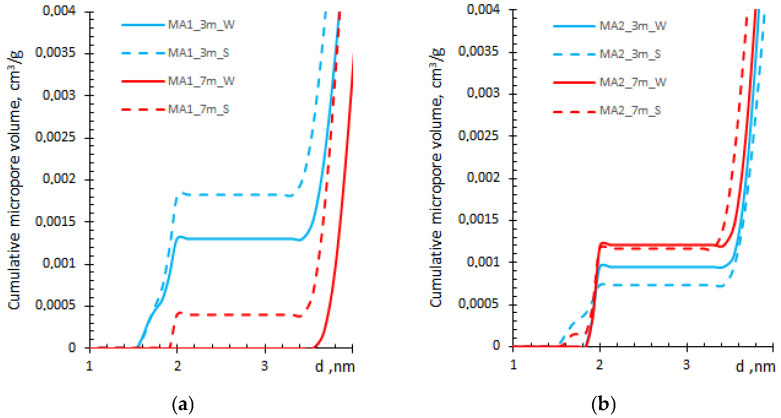
Comparison of cumulative micropore volume obtained by QSDFT method for MA1 (**a**) and MA2 (**b**) samples.

Usually, small gel pores (less than 3 nm) are underestimated by the BJH method (Figure 3a) [23], whereas the QSDFT method allows the determination of the cumulative micropore volume (Figure 4).

The QSDFT method showed a false minimum or no contribution from pores of 2–3 nm in width (Figure 3 and Figure 4), indicating the possible disagreement between the theoretical isotherm and the data [25], which can be overcome by selecting an appropriate pore model. Nevertheless, all selected kernels showed artefacts in the small-gel region, indicating the complexity of the pore system in the MK–ash–cement system.

The comparison of small gel pores of the tested compositions can be performed through the pore size distribution with pore volume evaluation by another, Horvath–Kawazoe (HK) approach, which relies on macroscopic, thermodynamic assumptions concerning the nature of confined adsorbate, and originally developed for the slit-shape micropores [42].

**Figure 5:**
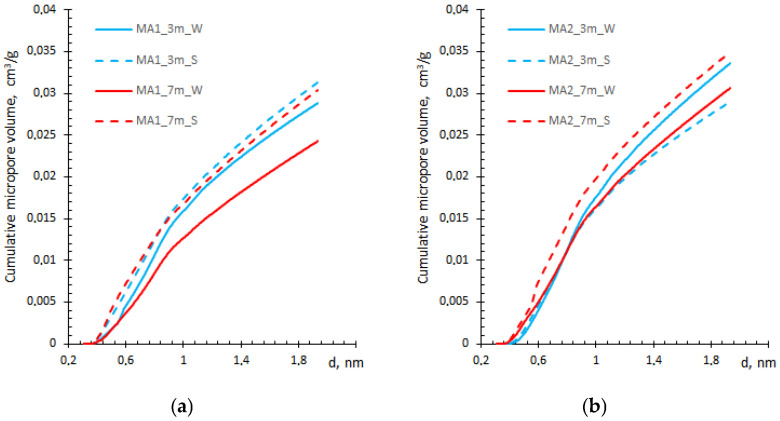
Comparison of cumulative micropore volume obtained by HK method for MA1 (**a**) and MA2 (**b**) samples.

The micropore volume derived from QSDFT (Figure 4) is lower by an order of magnitude than that obtained by the HK method (Figure 5), possibly due to a difference in the applied pore shape model. On the other hand, the data were comparable with micropore volume data obtained by the Dubinin–Radushkevich (DR) method (Table 2). Moreover, the HK data are similar to those presented in [30] for the synthesized pure C–S–H solids, suggesting the reliability of the selected HK method for the analysis of C–S–H structure in blended cement-based samples.

Figure 5 also shows that the ash type contributes to the micropore volume of the blended composite. The MA2 sample shows a higher overall pore volume than MA1, but the trend is opposite. The pore volume decreases with longer water exposure in both compositions; meanwhile, the prolonged curing in salt solution increases pore volume, mainly in the MA2 sample, with no significant effect in the MA1 sample (Figure 5).

**Figure 6:**
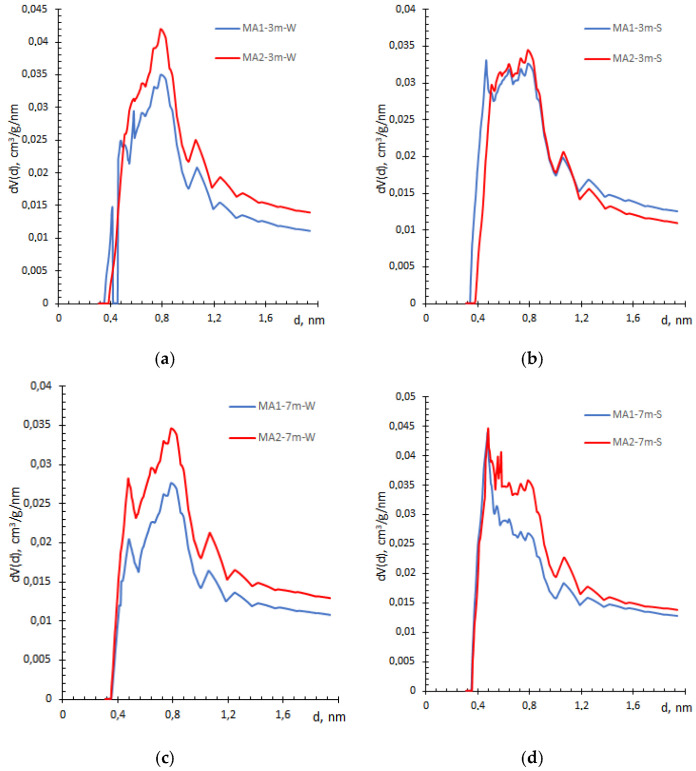
Comparison of pore width distribution obtained by HK method for MA1 and MA2 samples after exposure to lime water (**a**,**c**) and salt (**b**,**d**) for three and seven months.

Active carbon was used as the adsorbent in the HK model to calculate the pore size distribution. The distributions by HK approach (Figure 6) differ from those obtained by the QSDFT method (Figure 3) over the same pore size range. While the QSDFT method yields one or two modal pore size distributions, the HK method yields a cluster of peaks, with the highest concentration at 0.8 nm (Figure 5). According to X. Zhu et al. [30] synthesized C–S–H solids showed a similar trend, with a sharp peak in the range of 0.7 to 0.9 nm. In contrast, in samples exposed to the salt solution, the peak becomes less well-defined and narrower over time, implying a damaging effect of the curing solution on the C–S–H structure (Figure 6d). Such an effect is less obvious in QSDFT method (Figure 3).

Comparison of data obtained by HK and QSDFT methods indicates that the gel pores of the C–S–H structure are more sensitive to aggressive solution attack than interhydrate or mesopores (less than 10 nm). On the other hand, according to the findings presented in [24,30], variations in specific surface area, calculated by the BET method, provide insight into changes in the C–S–H microporosity. Therefore, the micropore surface area was calculated using the Dubinin–Radushkevich (DR) approach and is summarised in Table 1. Indeed, surface area data obtained by both methods show a linear correlation, thus confirming that the selected sample-drying method, operational temperature, and procedure selected in this study were appropriate for assessing the surface area of C–S–H gel pores.

### 3.4. Surface Fractal Dimension

The geometric topography of the surface structure of many solids can be characterized by the fractal dimension *D*, which is a kind of roughness exponent. The parameter *D* can be calculated using the modified Frenkel–Halsey–Hill (FHH) or Neimark–Kiselev (NK) method.

The FHH method is based on multilayer adsorption. P.J. Pomonis and E.T. Tsaousi [43] mentioned that when the van der Waals attraction is the predominant force between the solid and adsorbed film, the FHH isotherm obtains the form (Equation (1))

(1) $$log\left(\frac{V}{V_{m}}\right)=const+\left(\frac{D-3}{3}\right)\cdot \left[log\;log\left(\frac{p_{0}}{p}\right)\right]$$

When the surface tension between the liquid and gas becomes more important, the FHH isotherm is written in the form (Equation (2))

(2) $$log\left(\frac{V}{V_{m}}\right)=const+\left(D-3\right)\cdot \left[log\;log\left(\frac{p_{0}}{p}\right)\right]$$

In the NK method, the relationship between the area of the solid/liquid film interface and the average radius of the curvature of this interface is based on the thermodynamic fractal isotherm [43], which equation is (Equation (3))

(3) $$ln\left(S\right)=const-(D-2)\cdot ln\left(r\right)$$

where *V* is the adsorbed volume, *V_m_* is the volume of the monolayer, *p*_0_ and *p* are the saturated and equilibrium pressure of the adsorbate, *D* is the fractal dimension, *S* is the area of the solid–fluid interface, *r* is a curvature radius, and const. is a constant obtained from the ordinate intercept for a fitting plot of *log*(*V*/*V*_m_) vs. *log*(*log*(*p*_0_/*p*)) and *log*(*S*) vs. *log*(*r*).

The main difference between these methods is that the FHH model calculates D based on the adsorption film volume, whereas the NK method uses the adsorption film’s specific surface area. Data obtained by both methods are summarised in Table 3. When *D* equals 2, the surface is considered regular and smooth. However, for an irregular (real) surface, *D* may vary between 2 and 3, indicating the degree of surface roughness or porous structure, which increases with increasing *D*.

Data show that the *D* value calculated by the NK method exceeds 3 (Table 3), thus indicating the limitations of this method, especially for the characterisation of samples exposed to salt solution. It is worth noting that, despite the high correlation coefficient, the *D* value exceeds 3 in all MA2 sets, implying significant disturbance of the C–S–H pore structure. Other researchers also found the overestimated values by applying the NK method for cement-based materials [30,44,45].

On the contrary, the FHH model is more sensitive to small pore distribution; thus, a fractal dimension is in the 2.43–2.65 range, typical for C–S–H gel pores [44,46]. Although the values are close, the MA2 set shows slightly higher *D* values, suggesting greater roughness or a larger number of pores with varying widths. It correlates well with HK pore size distribution, especially for samples exposed to salt solution (Figure 6c,d and Figure 4). The correlations between DR micropore area (Table 2) and fractal dimension *D* are also seen as the minimum and the maximum values corresponding to the same compositions MA1-7m-W and MA2-7m-S, respectively. However, these results contradict statements that the increasing fractal dimension is a result of the physical packing of hydrates [44]. According to data in Table 3, the impact of the exposure environment on the *D* increment is obvious, while exposure duration to the aggressive solution contributes to the destruction of the C–S–H structure (Table 3).

## 4. Concluding Remarks

The application of the NAD method provides useful information for investigating the C–S–H structure when appropriate sample-drying methods and measurement parameters are used. The BJH method, generally used for pore size distribution, demonstrates limitations in the characterisation of C–S–H gel pore structure (Figure 3). In contrast, the DFT approach is rarely applied, even though it provides more comprehensive information, especially in the pore range that corresponds to C–S–H. Data provided by others [9,24,30] are comparable to those presented in this study, confirming the significance of the routine BET model for cement-based compositions, as evidenced by the type IV isotherms, which exhibit an unrestricted monolayer–multilayer zone (Figure 2).

A wide range of methods was applied to the NAD isotherms to evaluate the impact of exposure to an aggressive solution on the C–S–H structure of cement-based compositions blended with two types of ash. Regardless of ash type, both compositions showed changes in mesoporosity or in the C–S–H gel pore range, as evidenced by QSDFT calculations under impact of aggressive solution. The distinctions in microporosity (pores less than 2 nm) were assessed using the HK approach. HK and fractal dimension (FHH) results revealed that the C–S–H structure of composition with the incorporation of ash No. 2 from the Auvere plant [38] (MA2 set) was more deteriorated after the exposure to the sulfate–chloride solution, especially for a longer duration.

Overall, the findings of this study extend those presented in [24,29,30,45,47] by confirming that specific surface area calculated by the BET method provides quantitative information on the larger portion of C–S–H pores. However, the DR method should be applied to micropore volume evaluation, as it showed a correlation with the pore surface roughness characterised by the fractal dimension calculated by the FHH method.

## Figures and Tables

**Table 1:** Sample sets and notations.

Notations	Metakaolin	Ash Type		Curing Duration		Curing Medium	
		No. 1	No. 2	Months (m)		Lime Water	Salt Solution
MA1_Ref	M	A1		1 m		W	
MA2_Ref	M		A2		1 m	W	
MA1-3m-W	M	A1		3 m		W	
MA2-3m-W	M		A2		3 m	W	
MA1-3m-S	M	A1		3 m			S
MA2-3m-S	M		A2		3 m		S
MA1-7m-W	M	A1		7 m		W	
MA2-7m-W	M		A2		7 m	W	
MA1-7m-S	M	A1		7 m			S
MA2-7m-S	M		A2		7 m		S

**Table 2:** Parameters obtained from nitrogen physisorption measurements. Data for the reference material are taken from [38].

Sample	Ref. [38]	Curing Medium—Lime Water			Curing Medium—Salt Solution		
		3 Months [38]	7 Months	Change, %	3 Months	7 Months	Change, %
Total specific volume of pores less than 200 nm at *p*/*p*_0_ = 0.99, cm^3^/g							
MA1	0.1625	0.2126	0.1983	−7%	0.2010	0.2225	+11%
MA2	0.1926	0.2125	0.2163	+2%	0.1911	0.2112	+11%
Average pore diameter, nm							
MA1	21.93	10.58	10.98	+4%	9.07	10.12	+12%
MA2	11.86	8.75	9.77	+12%	9.50	8.55	−10%
Specific surface area (BET method) m^2^/g							
MA1	29.64	80.38	72.22	−10%	88.67	87.96	−1%
MA2	64.94	97.18	88.53	−9%	80.46	98.82	+23%
Micropore area (DR method), m^2^/g							
MA1	24.85	63.41	50.47	−20%	67.44	60.70	−10%
MA2	55.27	66.68	67.56	+1%	63.74	74.87	+18%
Micropore volume (DR method), cm^3^/g							
MA1	0.009	0.023	0.018	−22%	0.024	0.022	−8%
MA2	0.020	0.024	0.024	0%	0.023	0.027	+17%

Brunauer–Emmett–Teller (BET) method; Dubinin–Radushkevich (DR) method.

**Table 3:** Surface fractal dimension of MA1 and MA2 samples calculated from NK and FHH methods after curing in different environments.

Samples	NK Method			FHH Method			
	Fractal Dimension (*D*)			Fractal Dimension (*D*)			
	Slope (*h*)	*D = 2 − h*	*R* ^2^	Slope (*h*)	*D = 3(h + 1) (Equation (1))*	*D = 3 + h (Equation (2))*	*R* ^2^
1 month in lime water (reference samples)							
MA1	−0.735	2.7353	0.984	−0.567	*h* < −0.33	2.4328	0.998
MA2	−0.967	2.9672	0.994	−0.431	*h* < −0.33	2.5687	0.986
3 months in lime water							
MA1	−0.973	2.9726	0.994	−0.395	*h* < −0.33	2.6051	0.987
MA2	−1.121	3.1211	0.992	−0.371	*h* < −0.33	2.6288	0.977
7 months in lime water							
MA1	−0.949	2.9493	0.997	−0.400	*h* < −0.33	2.5997	0.988
MA2	−1.007	3.0068	0.994	−0.371	*h* < −0.33	2.6286	0.982
3 months in salt solution							
MA1	−1.050	3.0499	0.997	−0.366	*h* < −0.33	2.6343	0.981
MA2	−1.069	3.0692	0.994	−0.385	*h* < −0.33	2.6146	0.980
7 months in salt solution							
MA1	−1.002	3.0023	0.997	−0.381	*h* < −0.33	2.6149	0.981
MA2	−1.131	3.1306	0.995	−0.351	*h* < −0.33	2.6487	0.972

## Data Availability

The original contributions presented in this study are included in the article. Further inquiries can be directed to the corresponding author.
